# Supplementary material for: Real-World Use and Effectiveness of Carfilzomib Plus Dexamethasone in Relapsed/Refractory Multiple Myeloma in Europe
Source: Cancers (Basel). 2022 Oct 28;14(21):5311. doi: 10.3390/cancers14215311 (PMC9657308; doi:10.3390/cancers14215311)
Supplement: Supplementary file 1 [file cancers-14-05311-s001.zip › cancers-1973333-supplementary.pdf]

SUPPLEMENTAL DIGITAL CONTENT

Supplementary Table S1. Baseline disease and patient characteristics for lenalidomide-exposed patients by line of therapy.

	**Lenalidomide-Exposed: Refractory**			**Lenalidomide-Exposed: Not Refractory**		
	**2L/3L (n = 46)**	**4L+ (n = 105)**	**Overall (n = 151)**	**2L/3L (n = 12)**	**4L+ (n = 22)**	**Overall (n = 34)**
**Sex**						
Male	22 (47.8)	56 (53.3)	78 (51.7)	5 (41.7)	13 (59.1)	18 (52.9)
**Age at carfilzomib initiation**						
Mean, years (SD)	69.5 (7.8)	68.2 (8.3)	68.6 (8.1)	70.6 (7.1)	69.1 (6.5)	69.6 (6.6)
Median (min, max), years	70.0 (45, 83)	69.0 (50, 87)	70.0 (45, 87)	71.0 (56, 84)	70.5 (53, 78)	71.0 (53, 84)
< 65 years	9 (19.6)	34 (32.4)	43 (28.5)	3 (25.0)	6 (27.3)	9 (26.5)
65–74 years	26 (56.5)	46 (43.8)	72 (47.7)	7 (58.3)	12 (54.5)	19 (55.9)
≥ 75 years	11 (23.9)	25 (23.8)	36 (23.8)	2 (16.7)	4 (18.2)	6 (17.6)
**ISS stage at carfilzomib initiation [a]**	8 (17.4)	26 (24.8)	34 (22.5)	4 (33.3)	11 (50.0)	15 (44.1)
I [b]	3 (37.5)	11 (42.3)	14 (41.2)	0 (0.0)	2 (18.2)	2 (13.3)
II [b]	1 (12.5)	8 (30.8)	9 (26.5)	2 (50.0)	2 (18.2)	4 (26.7)
III [b]	4 (50.0)	7 (26.9)	11 (32.4)	2 (50.0)	7 (63.6)	9 (60.0)

**Patients with ECOG PS reported at carfilzomib initiation**	23 (50.0)	71 (67.6)	94 (62.3)	9 (75.0)	14 (63.6)	23 (67.6)
0–1 [b]	20 (87.0)	59 (83.1)	79 (84.0)	7 (77.8)	12 (85.7)	19 (82.6)
2–3 [b]	3 (13.0)	12 (16.9)	15 (16.0)	2 (22.2)	2 (14.3)	4 (17.4)
4 [b]	0 (0.0)	0 (0.0)	0 (0.0)	0 (0.0)	0 (0.0)	0 (0.0)
**Patients with derived frailty score at carfilzomib initiation [c]**	23 (50.0)	71 (67.6)	94 (62.3)	9 (75.0)	14 (63.6)	23 (67.6)
Fit (0) [b]	6 (26.1)	14 (19.7)	20 (21.3)	1 (11.1)	1 (7.1)	2 (8.7)
Intermediate (1) [b]	8 (34.8)	22 (31.0)	30 (31.9)	2 (22.2)	5 (35.7)	7 (30.4)
Frail (≥ 2) [b]	9 (39.1)	35 (49.3)	44 (46.8)	6 (66.7)	8 (57.1)	14 (60.9)

Data presented as n (%) unless stated otherwise. [a] Calculated from collected laboratory values. [b] Percentage is relative to the number of patients with data. [c] Patients with frailty score sums of 0, 1 or ≥ 2 were classified as fit, intermediate or frail, respectively. 2L = second line; 3L = third line; 4L+ = fourth or later lines; ECOG PS = Eastern Cooperative Oncology Group performance status; IMWG = International Myeloma Working Group; ISS = International Staging System.

Supplementary Table S2. Treatment history for the lenalidomide-exposed subgroup.

	Lenalidomide-Exposed: Refractory			Lenalidomide-Exposed: Not Refractory		
	**2L/3L (n = 46)**	**4L+ (n = 105)**	**Overall (n = 151)**	**2L/3L (n = 12)**	**4L+ (n = 22)**	**Overall (n = 34)**
**Number of prior lines of therapy**						
Median (min, max)	2.0 (1, 2)	4.0 (3, 10)	3.0 (1, 10)	2.0 (1, 2)	4.5 (3, 9)	3.0 (1, 9)
**Type of previous therapy**						
**PI** [a]	43 (93.5)	104 (99.0)	147 (97.4)	11 (91.7)	22 (100.0)	33 (97.1)
Bortezomib	43 (93.5)	103 (98.1)	146 (96.7)	11 (91.7)	21 (95.5)	32 (94.1)
Ixazomib	1 (2.2)	12 (11.4)	13(8.6)	2 (16.7)	2 (9.1)	4 (11.8)
Carfilzomib	0 (0.0)	6 (5.7)	6 (4.0)	0 (0.0)	3 (13.6)	3 (8.8)
**IMiD** [a]	46 (100.0)	105 (100.0)	151 (100.0)	12 (100.0)	22 (100.0)	34 (100.0)
Lenalidomide	46 (100.0)	105 (100.0)	151 (100.0)	12 (100.0)	22 (100.0)	34 (100.0)
Thalidomide	9 (19.6)	42 (40.0)	51 (33.8)	2 (16.7)	9 (40.9)	11 (32.4)
Pomalidomide	4 (8.7)	60 (57.1)	64 (42.4)	0 (0.0)	7 (31.8)	7 (20.6)
**Monoclonal antibody**	8 (17.4)	54 (51.4)	62 (41.1)	1 (8.3)	11 (50.0)	12 (35.3)
Daratumumab	7 (15.2)	49 (46.7)	56 (37.1)	1 (8.3)	11 (50.0	12 (35.3)
Isatuximab	1 (2.2)	2 (1.9)	3 (2.0)	0 (0.0)	0 (0.0)	0 (0.0)

**Previous HSCT**	16 (34.8)	63 (60.0)	79 (52.3)	5 (41.7)	13 (59.1)	18 (52.9)
**Refractory to any previous treatment line [b]**						
**Single-class refractory**	22 (47.8)	19 (18.1)	41 (27.2)	3 (25.0)	8 (36.4)	11 (32.4)
IMiD	22 (47.8)	19 (18.1)	41 (27.2)	0 (0.0)	1 (4.5)	1 (2.9)
PI	0 (0.0)	0 (0.0)	0 (0.0)	2 (16.7)	4 (18.2)	6 (17.6)
Anti-CD38	0 (0.0)	0 (0.0)	0 (0.0)	1 (8.3)	3 (13.6)	4 (11.8)
**Double-class refractory**	18 (39.1)	47 (44.8)	65 (43.0)	0 (0.0)	6 (27.3)	6 (17.6)
PI + IMiD	17 (37.0)	35 (33.3)	52 (34.4)	0 (0.0)	2 (9.1)	2 (5.9)
Anti-CD38 + IMiD	1 (2.2)	12 (11.4)	13 (8.6)	0 (0.0)	1 (4.5)	1 (2.9)
Anti-CD38 + PI	0 (0.0)	0 (0.0)	0 (0.0)	0 (0.0)	3 (13.6)	3 (8.8)
**Triple-class refractory**	6 (13.0)	39 (37.1)	45 (29.8)	0 (0.0)	3 (13.6)	3 (8.8)
Anti-CD38 + PI + IMiD	6 (13.0)	39 (37.1)	45 (29.8)	0 (0.0)	3 (13.6)	3 (8.8)
**Not refractory**	0 (0.0)	0 (0.0)	0 (0.0)	9 (75.0)	5 (22.7)	14 (41.2)

Data presented as n (%) unless stated otherwise. [a] Patients may have received more than one drug within a given drug class. Hence, the total numbers reported for each drug class may be smaller than the sum of the individual values of each drug within that class. [b] A patient was classified as refractory to a drug by IMWG definition if they met at least one of the three following criteria: best response to any regimen containing the drug was either stable or progressive disease; reason the treatment was stopped was progression in any regimen containing the drug; date of relapse/progression was after the start date and within 60 days (inclusive) after the stop date of the drug in any regimen containing the drug. 2L = second line; 3L = third line; 4L+ = fourth or later lines; HSCT = hematopoietic stem cell transplant; IMiD = immunomodulatory drug; IMWG = International Myeloma Working Group; PI = proteasome inhibitor.

Supplementary Table S3. Baseline disease and patient characteristics for patients with anti-CD38 mAb-refractory disease by line of therapy.

	**Anti-CD38 mAb-Refractory**		
	**2L/3L (n = 9)**	**4L+ (n = 62)**	**Overall (n = 71)**
**Sex**			
Male	6 (66.7)	41 (66.1)	47 (66.2)
**Age at carfilzomib initiation**			
Mean, years (SD)	70.2 (7.9)	67.9 (8.9)	68.2 (8.7)
Median (min, max), years	70.0 (56, 80)	68.0 (50, 87)	69.0 (50, 87)
< 65 years	2 (22.2)	24 (38.7)	26 (36.6)
65–74 years	4 (44.4)	22 (35.5)	26 (36.6)
≥ 75 years	3 (33.3)	16 (25.8)	19 (26.8)
**ISS stage at carfilzomib initiation** [a]	1 (11.1)	16 (25.8)	17 (23.9)
I [b]	0 (0.0)	5 (31.3)	5 (29.4)
II [b]	1 (100.0)	3 (18.8)	4 (23.5)
III [b]	0 (0.0)	8 (50.0)	8 (47.1)
**Patients with ECOG PS reported at carfilzomib initiation**	6 (66.7)	41 (66.1)	47 (66.2)
0–1 [a]	5 (83.3)	33 (80.5)	38 (80.9)
2–3 [a]	1 (16.7)	8 (19.5)	9 (19.1)
4 [a]	0 (0.0)	0 (0.0)	0 (0.0)

**Patients with derived frailty score at carfilzomib initiation** [c]	6 (66.7)	41 (66.1)	47 (66.2)
Fit (0) [a]	1 (16.7)	6 (14.6)	7 (14.9)
Intermediate (1) [a]	3 (50.0)	11 (26.8)	14 (29.8)
Frail (≥ 2) [a]	2 (33.3)	24 (58.5)	26 (55.3)

Data presented as n (%) unless stated otherwise. The full analysis set includes all patients enrolled in the study whose end of study reason is not 'Decision by Sponsor'. [a] Percentage is relative to the number of patients with data. [b] Calculated from collected laboratory values. [c] Patients with frailty score sums of 0, 1 or ≥ 2 were classified as fit, intermediate or frail, respectively.

2L = second line; 3L= third line; 4L+ = fourth or later lines; ECOG PS = Eastern Cooperative Oncology Group performance status; IMWG = International Myeloma Working Group; ISS = International Staging System; mAb = monoclonal antibody.

Supplementary Table S4. Treatment history for patients with anti-CD38 mAb-refractory disease by line of therapy.

	Anti-CD38 mAb-Refractory		
	**2L/3L (n = 9)**	**4L+ (n = 62)**	**Overall (n = 71)**
**Number of prior lines of therapy**			
Median (min, max)	2.0 (1, 2)	5.0 (3, 9)	4.0 (1, 9)
**Type of previous therapy**			
**PI** [a]	9 (100.0)	61 (98.4)	70 (98.6)
Bortezomib	9 (100.0)	60 (96.8)	69 (97.2)
Ixazomib	0 (0.0)	9 (14.5)	9 (12.7)
Carfilzomib	0 (0.0)	5 (8.1)	5 (7.0)
**IMiD** [a]	8 (88.9)	62 (100.0)	70 (98.6)
Lenalidomide	8 (88.9)	61 (98.4)	69 (97.2)
Thalidomide	3 (33.3)	19 (30.6)	22 (31.0)
Pomalidomide	1 (11.1)	40 (64.5)	41 (57.7)
**mAb**	9 (100.0)	62 (100.0)	71 (100.0)

Daratumumab	8 (88.9)	60 (96.8)	68 (95.8)
Isatuximab	1 (11.1)	2 (3.2)	3 (4.2)
**Previous HSCT**	4 (44.4)	36 (58.1)	40 (56.3)
**Anti-CD38 mAb given as maintenance therapy**	0 (0.0)	0 (0.0)	0 (0.0)
**Induction included an anti-CD38 mAb**	0 (0.0)	0 (0.0)	0 (0.0)
**Induction did not include an anti-CD38 mAb**	0 (0.0)	0 (0.0)	0 (0.0)
**Anti-CD38 mAb not given as maintenance therapy**	9 (100.0)	62 (100.0)	71 (100.0)
Monotherapy	2 (22.2)	35 (56.5)	37 (52.1)
Combination therapy	7 (77.8)	30 (48.4)	37 (52.1)
**Refractory to any previous treatment line [b]**			
**Single-class refractory**	2 (22.2)	3 (4.8)	5 (7.0)
PI	0 (0.0)	0 (0.0)	0 (0.0)
IMiD	0 (0.0)	0 (0.0)	0 (0.0)
Anti-CD38 mAb	2 (22.2)	3 (4.8)	5 (7.0)

**Double-class refractory**	1 (11.1)	16 (25.8)	17 (23.9)
Anti-CD38 mAb + IMiD	1 (11.1)	13 (21.0)	14 (19.7)
Anti-CD38 mAb + PI	0 (0.0)	3 (4.8)	3 (4.2)
PI + IMiD	0 (0.0)	0 (0.0)	0 (0.0)
**Triple-class refractory**	6 (66.7)	43 (69.4)	49 (69.0)
Anti-CD38 mAb + PI + IMiD	6 (66.7)	43 (69.4)	49 (69.0)
**Not refractory**	0 (0.0)	0 (0.0)	0 (0.0)

Data presented as n (%) unless stated otherwise. [a] Patients may have received more than one drug within a given drug class. Hence, the total numbers reported for each drug class may be smaller than the sum of the individual values of each drug within that class. [b] A patient was classified as refractory to a drug by IMWG definition if they met at least one of the three following criteria: best response to any regimen containing the drug was either stable or progressive disease; reason the treatment was stopped was progression in any regimen containing the drug; date of relapse/progression was after the start date and within 60 days (inclusive) after the stop date of the drug in any regimen containing the drug. 2L = second line; 3L = third line; 4L+ = fourth or later lines; HSCT = hematopoietic stem cell transplant; IMiD = immunomodulatory drug; IMWG = International Myeloma Working Group; mAb = monoclonal antibody; PI = proteasome inhibitor.

Supplementary Table S5. Summary of AEs – overall and by line of therapy for lenalidomide-exposed patients.

	**Lenalidomide-Exposed: Refractory**			**Lenalidomide-Exposed: Not Refractory**		
	**2L/3L (n = 46)**	**4L+ (n = 105)**	**Overall (n = 151)**	**2L/3L (n = 12)**	**4L+ (n = 22)**	**Overall (n = 34)**
**All CTCAE grade 3 and above TEAEs**	19 (41.3)	57 (54.3)	76 (50.3)	4 (33.3)	10 (45.5)	14 (41.2)
SAEs	14 (30.4)	45 (42.9)	59 (39.1)	3 (25.0)	10 (45.5)	13 (38.2)
AEs leading to carfilzomib discontinuation	1 (2.2)	20 (19.0)	21 (13.9)	0 (0.0)	5 (22.7)	5 (14.7)
Fatal AEs	3 (6.5)	11 (10.5)	14 (9.3)	1 (8.3)	1 (4.5)	2 (5.9)
**All CTCAE grade 3 and above treatment-related TEAEs**	8 (17.4)	29 (27.6)	37 (24.5)	3 (25.0)	4 (18.2)	7 (20.6)
SAEs	6 (13.0)	18 (17.1)	24 (15.9)	2 (16.7)	4 (18.2)	6 (17.6)
AEs leading to carfilzomib discontinuation	0 (0.0)	12 (11.4)	12 (7.9)	0 (0.0)	2 (9.1)	2 (5.9)
Fatal AEs [a]	0 (0.0)	2 (1.9)	2 (1.3)	0 (0.0)	0 (0.0)	0 (0.0)

**Most common treatment-related TEAEs by SOC (reported in ≥ 5% of any subgroup or overall) and classified by HLGT [b] or PT [c]**						
**Blood and lymphatic system disorders**	**3 (6.5)**	**12 (11.4)**	**15 (9.9)**	**1 (8.3)**	**1 (4.5)**	**2 (5.9)**
Anemia [c]	2 (4.3)	5 (4.8)	7 (4.6)	0 (0.0)	0 (0.0)	0 (0.0)
Febrile neutropenia [c]	0 (0.0)	1 (1.0)	1 (0.7)	0 (0.0)	0 (0.0)	0 (0.0)
Neutropenia [c]	1 (2.2)	3 (2.9)	4 (2.6)	0 (0.0)	0 (0.0)	0 (0.0)
Thrombocytopenia [c]	3 (6.5)	7 (6.7)	10 (6.6)	1 (8.3)	1 (4.5)	2 (5.9)
**Respiratory, thoracic and mediastinal disorders**	**3 (6.5)**	**6 (5.7)**	**9 (6.0)**	**0 (0.0)**	**0 (0.0)**	**0 (0.0)**
Dyspnea [c]	2 (4.3)	2 (1.9)	4 (2.6)	0 (0.0)	0 (0.0)	0 (0.0)
Lower respiratory tract disorders (excluding obstruction and infection) [b]	1 (2.2)	3 (2.9)	4 (2.6)	0 (0.0)	0 (0.0)	0 (0.0)
Lung disorder [c]	0 (0.0)	1 (1.0)	1 (0.7)	0 (0.0)	0 (0.0)	0 (0.0)
**Cardiac disorders**	**0 (0.0)**	**9 (8.6)**	**9 (6.0)**	**0 (0.0)**	**1 (4.5)**	**1 (2.9)**
Cardiac arrhythmias [b]	0 (0.0)	2 (1.9)	2 (1.3)	0 (0.0)	1 (4.5)	1 (2.9)
Coronary artery disorders [b]	0 (0.0)	1 (1.0)	1 (0.7)	0 (0.0)	0 (0.0)	0 (0.0)
Heart failures [b]	0 (0.0)	6 (5.7)	6 (4.0)	0 (0.0)	0 (0.0)	0 (0.0)
**Renal and urinary disorders**	**0 (0.0)**	**2 (1.9)**	**2 (1.3)**	**1 (8.3)**	**1 (4.5)**	**2 (5.9)**
Acute kidney injury [c]	0 (0.0)	1 (1.0)	1 (0.7)	1 (8.3)	0 (0.0)	1 (2.9)
Renal failure [c]	0 (0.0)	1 (1.0)	1 (0.7)	0 (0.0)	1 (4.5)	1 (2.9)
**Vascular disorders**	**2 (4.3)**	**6 (5.7)**	**8 (5.3)**	**0 (0.0)**	**1 (4.5)**	**1 (2.9)**
Hypertension [c]	2 (4.3)	6 (5.7)	8 (5.3)	0 (0.0)	0 (0.0)	0 (0.0)

Hypertensive crisis [c]	0 (0.0)	0 (0.0)	0 (0.0)	0 (0.0)	1 (4.5)	1 (2.9)
Hypotension [c]	0 (0.0)	1 (1.0)	1 (0.7)	0 (0.0)	0 (0.0)	0 (0.0)
**Infections and infestations**	**1 (2.2)**	**4 (3.8)**	**5 (3.3)**	**1 (8.3)**	**1 (4.5)**	**2 (5.9)**
Ancillary infectious topics [b]	0 (0.0)	0 (0.0)	0 (0.0)	1 (8.3)	0 (0.0)	1 (2.9)
Bacterial infectious disorders [b]	1 (2.2)	1 (1.0)	2 (1.3)	0 (0.0)	0 (0.0)	0 (0.0)
Infections, unspecified [b]	0 (0.0)	3 (2.9)	3 (2.0)	0 (0.0)	1 (4.5)	1 (2.9)

Data presented as n (%). n represents the number of patients who experienced one or more AEs. Patients were counted only once for each PT, HLGT or SOC level. The total number at the SOC level may be lower than the sum of the individual numbers reported at HLGT or PT level, because one patient could experience multiple events. AEs were coded using MedDRA version 23.0 and graded using NCI-CTCAE version 4.03. [a] Fatal treatment-related TEAEs were due to cardiac disorders (one fatal cardiac arrhythmia; one fatal coronary artery disorder). [b] Treatment-related TEAE HLGT classification. [c] Treatment-related TEAE PT classification. 2L = second line; 3L = third line; 4L+ = fourth or later lines; AE = adverse event; CTCAE = Common Terminology Criteria for Adverse Events; HLGT = High-Level Group Term; PT = Preferred-Term; SAE = serious AE; SOC = System Organ Class; TEAE = treatment-emergent adverse event.

Supplementary Table S6. Summary of AEs – overall and by line of therapy for patients with anti-CD38 mAb-refractory disease.

	**Anti-CD38 mAb-Refractory**		
	**2L/3L (n = 9)**	**4L+ (n = 62)**	**Overall (n = 71)**
**All CTCAE grade 3 and above TEAEs**	4 (44.4)	39 (62.9)	43 (60.6)
SAEs	4 (44.4)	33 (53.2)	37 (52.1)
AEs leading to discontinuation of carfilzomib	0 (0.0)	16 (25.8)	16 (22.5)
Fatal AEs	0 (0.0)	8 (12.9)	8 (11.3)
**All CTCAE grade 3 and above treatment-related TEAEs**	0 (0.0)	17 (27.4)	17 (23.9)
SAEs	0 (0.0)	12 (19.4)	12 (16.9)
AEs leading to discontinuation of carfilzomib	0 (0.0)	7 (11.3)	7 (9.9)
Fatal AEs[a]	0 (0.0)	1 (1.6)	1 (1.4)
**Most common treatment-related TEAEs by SOC (reported in ≥ 5% of any subgroup or overall) and classified by HLGT [b] or PT [c]**			
**Blood and lymphatic system disorders**	**0 (0.0)**	**6 (9.7)**	**6 (8.5)**
Anemia [c]	0 (0.0)	3 (4.8)	3 (4.2)
Neutropenia [c]	0 (0.0)	1 (1.6)	1 (1.4)
Thrombocytopenia [c]	0 (0.0)	4 (6.5)	4 (5.6)
**Cardiac disorders**	**0 (0.0)**	**6 (9.7)**	**6 (8.5)**
Cardiac arrhythmia [b]	0 (0.0)	1 (1.6)	1 (1.4)

Coronary artery disorders [b]	0 (0.0)	1 (1.6)	1 (1.4)
Heart failures [b]	0 (0.0)	4 (6.5)	4 (5.6)
**Vascular disorders**	**0 (0.0)**	**4 (6.5)**	**4 (5.6)**
Hypertension [c]	0 (0.0)	3 (4.8)	3 (4.2)
Hypertensive crisis [c]	0 (0.0)	1 (1.6)	1 (1.4)
**Infections and infestations**	**0 (0.0)**	**4 (6.5)**	**4 (5.6)**
Bacterial infectious disorders [b]	0 (0.0)	1 (1.6)	1 (1.4)
Infections, unspecified [b]	0 (0.0)	3 (4.8)	3 (4.2)

Data presented as n (%). n represents the number of patients who experienced one or more AEs. Patients were counted only once for each PT, HLGT or SOC level. The total number at the SOC level may be lower than the sum of the individual numbers reported at HLGT or PT level, because one patient could experience multiple events. AEs were coded using MedDRA version 23.0 and graded using NCI-CTCAE version 4.03. [a] Fatal treatment-related TEAE was due to a coronary artery disorder (one fatal acute myocardial infarction). [b] Treatment-related TEAE HLGT classification. [c] Treatment-related TEAE PT classification. 2L = second line; 3L = third line; 4L+ = fourth or later lines; AE = adverse event; CTCAE = Common Terminology Criteria for Adverse Events; HLGT = High-Level Group Term; mAb = monoclonal antibody; PT = Preferred-Term; SAE = serious AE; SOC = System Organ Class; TEAE = treatment-emergent adverse event.
